# Veillonella parvula periorbital cellulitis: an unusual pathogen causing a common clinical sign

**DOI:** 10.3205/oc000106

**Published:** 2019-05-29

**Authors:** Liesbeth Wellens, Ingele Casteels, Marc Huygens

**Affiliations:** 1 Department of Ophthalmology, University Hospitals KU Leuven, Belgium; 2 Department of Ophthalmology, AZ Sint-Lucas Brugge, Bruges, Belgium

## Abstract

We describe the case of a one-year-old boy who presented at the emergency department with a sudden onset of fulminant edema of the right eyelid. He had been suffering from a varicella zoster virus (VZV) infection for 5 days. A secondary bacterial infection of varicella skin lesions was suspected. Computed tomography of the orbit revealed pronounced superficial soft tissue inflammation of the right periorbit, without intraorbital extension. There was a spontaneous rupture of the right upper eyelid and a culture of the released fluid grew the anaerobic organism *Veillonella parvula*. The patient was treated with clindamycin for 2 months and made a slow, yet full recovery.

## Introduction

*Veillonella parvula*, a strictly anaerobic gram-negative coccus, is described as a commensal of the oropharynx, gastrointestinal tract and vaginal flora in humans. It is rarely described as an infectious cause although the literature reports a few cases concerning periodontitis, meningitis, endocarditis, osteomyelitis, pelvic, pulmonary and epidural abscesses, and life-threatening bacteraemia [1], [2], [3], [4], [5], [6], [7], [8], [9], [10].

In this case, the periorbital cellulitis is caused by *V. parvula* and assumed to be secondary to a varicella infection. A literature review on *V. parvula* pathogenicity is reported [1], [2], [3], [4], [5], [6], [7], [8], [9], [10], [11], [12].

To the best of our knowledge, this is the first case report describing an isolated periorbital cellulitis caused by *V. parvula*.

## Case description

**Figure 1:**
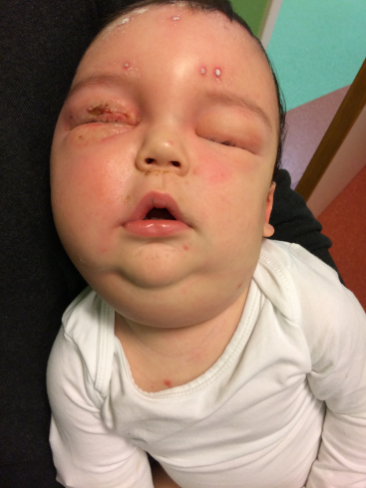
Bilateral eyelid edema on day 2 with spontaneous rupture of the right upper eyelid

A one-year-old boy presented to the emergency department with a sudden onset of fulminant edema of the right eyelid, making it impossible to open his right eye (Figure 1 (Fig. 1)). The ophthalmologic examination showed painful eyelid edema with conjunctival chemosis. Due to the extensive eyelid swelling, it wasn’t possible to examine pupillary light reflexes or ocular motility nor to perform a fundus examination. The boy had a temperature up to 39.1°C. Physical examination showed an alert patient, without neurological or meningeal signs, but with cutaneous varicella lesions spread over his entire body.

**Figure 2:**
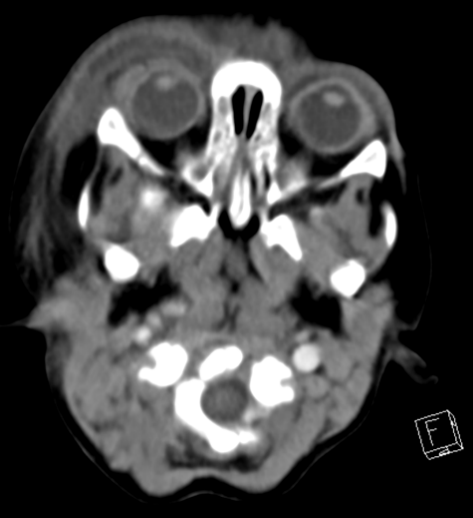
Computed tomography (axial scan) of the orbit showing extensive swelling of the right periorbital soft tissues and minimal swelling on the left side, without intraorbital involvement

Laboratory work up showed normal leukocytes (9.23 x 10^3^/µl), thrombocytopenia (platelets 67 x 10^3^/µl) and elevated CRP (52 mg/l). The boy hadn’t received his vaccination for varicella yet, which usually takes place between the age of 12 to 15 months. Clinical examination couldn’t exclude orbital cellulitis and revealed multiple enlarged lymph nodes in the neck region. There was a clinical suspicion of a secondary bacterial infection of the cutaneous varicella lesions around the eyelid. CT of the orbit revealed pronounced superficial soft tissue inflammation of the right periorbit without evidence of an intraorbital inflammation or abscess formation, cavernous sinus thrombosis or intracranial extension of the inflammation (Figure 2 (Fig. 2)). 

**Figure 3:**
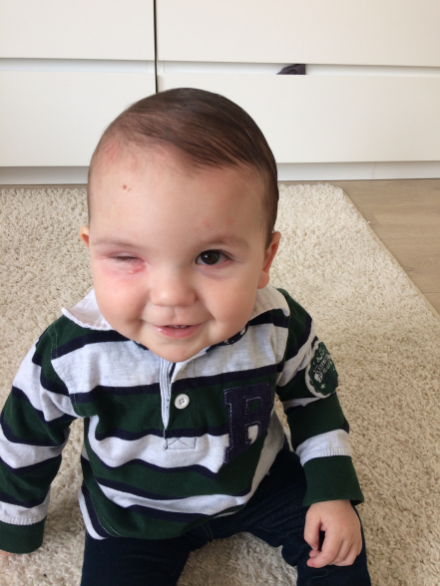
Persistent hardening of the eyelid after resolution of the edema with incomplete opening of the right eye after one month, obscuring the visual axis

The boy was hospitalized for a trial of intravenously antiviral (acyclovir) and antibiotic therapy (amikacin, flucloxacillin, and ceftriaxone). The antibiotic therapy was changed to clindamycin after 2 days due to poor clinical response with also increasing swelling of his left eyelid (Figure 1 (Fig. 1)). In immunocompetent children, VZV usually causes a benign infection without the need for systemic antiviral treatment, in this case the pediatrician decided to treat the varicella infection with intravenously acyclovir for 5 days (30 mg/kg/day in 3 divided doses). The laboratory markers of inflammation raised with a maximal leucocytosis of 23.8 x 10^3^/µl and a CRP level of 190 mg/l. There weren’t any clinical signs of sepsis. The peripheral blood count and culture of vesicular fluid obtained at the emergency department were negative. On the second day of admission, the right upper eyelid ruptured spontaneously (Figure 1 (Fig. 1)). We removed the overlying crusts and debris and took a swab of the underlying ulcer. Culture grew the anaerobic organism *Veillonella parvula*, without identifying any other bacterial or fungal organisms. Clindamycin was continued to cover anaerobe bacteria. The antibiogram showed resistance to penicillin, amoxicillin clavulanic acid and even metronidazole, but sensitivity to clindamycin and meropenem. The source of infection was most likely a superinfection of the varicella skin lesion. Clindamycin was given intravenously for 3 weeks with a good clinical response showing a slow but progressive decline of the eyelid edema. When the boy was discharged from the hospital, clindamycin was continued orally for another 4 weeks, resulting in a total antibiotic treatment duration of 7 weeks. The eyelid swelling with a hardened eyelid persisted for 4 months. Because of this, the boy was unable to fully open his eye in the first 4 months, obscuring the child’s visual axis and putting him at great risk for developing occlusion amblyopia (Figure 3 (Fig. 3)). The patient gained a full recovery with a complete resolution of the eyelid edema and a clear visual axis without need for surgical intervention. He is still in follow-up with a pediatric ophthalmologist for regular amblyopia screening and early treatment if necessary.

## Discussion

Periorbital or preseptal cellulitis of the eyelid is defined by an infection of the eyelid anterior to the orbital septum. Most often it’s caused by direct spread from a contiguous infected site, such as an adjacent sinusitis or dacryocystitis, but it can also be caused by primary inoculation through a minor trauma to the eyelid, such as an insect bite. In our patient, the periorbital cellulitis was most probably caused by superinfection of varicella skin lesions around the eyelid. Although varicella is generally considered a benign self-limiting disease in childhood, severe complications may occur requiring hospitalization. Bacterial superinfection is the most common complication, often caused by scratching. This can be the cause of superficial bacterial cellulitis, sometimes leading to a more severe necrotizing fasciitis, abscess formation and Varicella gangrenosa. Especially group A *streptococcus* are described as a potential cause and antibiotic therapy may be required [13]. In adults, although less common, VZV (primary infection or reactivation) is more severe with more severe complications, especially in immunocompromised patients. In this population, VZV can be associated with thrombosis of cerebral arteries and venous sinuses secondary to varicella induced hypercoagulable state [14], [15]. Sinus cavernous thrombosis, although very unlikely in our case, was excluded by CT.

In healthy and immunocompetent children, varicella is associated with low rates of morbidity and mortality and rarely requires antiviral therapy. Supportive care is normally sufficient for most of the children and trimming of the fingernails helps preventing bacterial superinfections caused by scratching. The decision whether to initiate systemic antiviral therapy in a patient with chickenpox will depend on the patient’s age, underlying medical conditions, and the risk of complications. Since the Food and Drug Administration (FDA) approved this vaccine in 1995 for use in people 1 year of age and older, the incidence of chickenpox has declined dramatically, reducing the need for antiviral therapy in the pediatric population. 

In our patient, *V. parvula* caused a severe bacterial preseptal cellulitis with an extensive clinical course. *V. parvula* infections are rarely described in the literature. It’s mainly considered as a non-pathogenic commensal organism, usually involved in polymicrobial processes, as it is the case with other anaerobic infections. This finding makes it difficult to investigate its pathogenicity. However, in our patient *V. parvula* presented as a single isolated organism and should therefore be considered as the true causative pathogen and treated according to the antibiogram. In the literature there is no consensus about the treatment recommendations due to limited reports on *Veillonella* pathogenic infections. *V. parvula* infection is reported to respond well to therapy with penicillin or metronidazole [11], [12]. In our case, the organism was resistant to both penicillin and metronidazole, emphasizing the importance of antimicrobial susceptibility testing and targeted antibiotic therapy. In the absence of orbital involvement, most of the periorbital cellulitis cases can be successfully treated with appropriate antibiotic therapy. It’s important to start the correct antibiotics as soon as possible to prevent intraorbital or intracranial extension. 

In young children, it’s even more important to treat preseptal cellulitis more aggressively so a quick recovery of the swelling can be achieved in order to prevent occlusion amblyopia in the future. Once a child is identified with an amblyopia, risk factor it’s of great importance that the child has regular check-ups with a pediatric ophthalmologist for a comprehensive examination.

## Conclusion

This is the first case report of periorbital cellulitis caused by *V. parvula* in a child with primary VZV infection and emphasises the importance of appropriate empiric antibiotic therapy in preseptal cellulitis. Therapy should be started immediately and modified based on clinical response, the interpretation of Gram’s stain, (repeated) culture, and antimicrobial susceptibility results. Atypical pathogens can cause common clinical signs of daily clinical interest and should not be forgotten in our differential diagnosis. 

## Notes

### Informed consent 

Parent/guardian consent was obtained for reproducing any pertinent patient specific information. 

### Competing interests

The authors declare that they have no competing interests.
